# Prevalence of Diamine Oxidase Enzyme (DAO) Deficiency in Subjects with Insomnia-Related Symptoms

**DOI:** 10.3390/jcm13164583

**Published:** 2024-08-06

**Authors:** Raquel López García, Júlia Ferrer-Garcia, Anna Sansalvador, Maria-Antonia Quera-Salva

**Affiliations:** AdSalutem Institute for Healthy Sleep, C/Mallorca 273, E-08008 Barcelona, Spainmaquera@adsalutem.healthcare (M.-A.Q.-S.)

**Keywords:** diamine oxidase enzyme, insomnia, sleep problems, genetic variants, single nucleotide polymorphism, histamine, *AOC1* gene

## Abstract

**Background**: To assess the prevalence of diamine oxidase (DAO) enzyme deficiency caused by single nucleotide polymorphisms (SNPs) of the *AOC1* gene in a sample of patients with symptoms of insomnia. **Methods**: A total of 167 adult patients (>18 years of age) with symptoms of insomnia attended a specialized institute for healthy sleep, in Barcelona (Spain), between May and November 2023, and underwent genotyping analysis of the four most relevant SNP variants, including c.691G>7 (rs2052129), c.47C>T (rs10156191), c.995C>T (rs1049742), and c.1990C>G (rs1049793). **Results**: Genetic DAO deficiency was present in 138 patients, with a prevalence rate of 82.6% (95% CI 76–88.1%). Difficulties in staying asleep were the most common complaints in 88% of patients followed by trouble falling asleep in 60.5%. More than half of patients suffered from insomnia symptoms every day. Also, 99.4% reported daytime consequences of insomnia, with fatigue (79.6%), mood changes (72.5%), and impaired concentration in 70.1%. When patients were grouped by DAO-score, which reflected the number of heterozygous and homozygous SNPs variants, the group with a DAO-score ≥ 4 vs. 1 showed higher percentages of insomnia-related symptoms, in particular, trouble staying asleep and early morning awakening. These two symptoms were also more common in the presence of the c.1990C>G (rs1049793) variant. **Conclusions**: This preliminary real-world study presents novel evidence of a potential link between a DAO enzyme deficiency of a genetic origin and clinical symptoms of insomnia, which may suggest the potential benefit of DAO supplementation to improve the quality of sleep in these subjects. The study was registered at ClinicalTrials.gov (NCT06488027).

## 1. Introduction

Insomnia is a common sleep disorder defined by the International Classification of Sleep Disorders-Third Edition (ICSD-3) as a single, chronic disorder characterized by sleep initiation or maintenance problems (despite the presence of adequate opportunity and circumstances for sleep) and daytime consequences, with a duration criterion of 3 months and a frequency criterion of at least three times per week [1]. It has been estimated that chronic insomnia is present in 6% of the population of Western industrialized countries [2,3], with large variations among European countries from a low prevalence of 5.7% in Germany up to 19% in France and 6.9% in the population of Spain [3,4]. However, approximately 30% of a variety of adult samples drawn from population-based studies reported one or more of the symptoms of insomnia: difficulty initiating sleep, difficulty maintaining sleep, waking up too early, and in some cases, a nonrestorative or poor quality of sleep [5]. Insomnia is a risk factor for multiple conditions, so that a better understanding and characterization of insomnia may contribute to tailoring individualized evaluation and treatment, as well as preventing other related diseases [3].

Diamine oxidase (DAO) is an enzyme encoded by the amine oxidase copper-containing 1 (*AOC1*) gene located in chromosome 7 (7q34-q36) in the human genome [6]. DAO is a secretory protein stored in plasma membrane vesicular structures and responsible for the degradation of extracellular histamine [7,8]. In mammals, DAO is mainly expressed in the small intestinal villi [9,10]. An impaired histamine degradation based on reduced DAO activity and the resulting exogenous histamine excess may cause numerous symptoms due to the ubiquitous distribution of histamine receptors in organs and tissues [11].

Deficiency in the DAO enzyme has a strong genetic component [12], but the prevalence of this in the general population remains undefined. In a recent large random population-based sample of 1051 healthy subjects in the framework of the West Sweden Asthma Study, DAO deficiency was reported in 44% of cases [13]. DAO activity is largely associated with single nucleotide polymorphisms (SNPs) of the *AOC1* gene, particularly the three most relevant variants in Caucasian individuals leading to a reduction in DAO enzyme activity are c.47C>T (rs10156191), c.995C>T (rs1049742), and c.1990C>G (rs1049793) [14,15]. The following frequencies (95% confidence interval [CI]) for these three *AOC1* gene variants among Spanish Caucasian individuals have been reported: rs10156191, 25.4% (20.16–30.58); rs1049742, 6.3% (3.42–9.26); and rs1049793, 30.6% (25.1–36.1) [14]. In addition, another SNP in the promoter region of the *AOC1* gene has been identified, c.691G>T (rs2052129) with a frequency of 41.7%, which has been associated with a decrease in DAO transcriptional activity [15].

Histamine intolerance due to DAO dysfunction may cause nonspecific functional gastrointestinal and extraintestinal symptoms with large variability and complex symptom combinations of digestive, cardiovascular, respiratory, cutaneous, and neurological manifestations [11]. In relation to the nervous system, a high rate of DAO deficiency in 87% of patients with proven diagnoses of migraines has been reported [16]. In a cohort of pediatric patients with attention deficit hyperactivity disorder (ADHD), the prevalence of having at least one minor dysfunctional allele was 78.5% [17], and in women with fibromyalgia, the prevalence of genetic DAO deficiency was 74.5% based on alterations in the four variants of the *AOC1* gene [18]. Sleep issues like insomnia can affect patients with these disorders [19,20,21]. Moreover, sleep problems are frequent complaints in subjects with DAO deficiency; however, as far as we are aware, DAO deficiency in subjects with insomnia symptoms has not been previously evaluated.

Therefore, the objective of the study was to determine the prevalence of DAO deficiency of a genetic origin in subjects who presented insomnia symptoms.

## 2. Materials and Methods

### 2.1. Design and Participants

A prospective study of the prevalence of DAO enzyme deficiency in patients with insomnia-related symptoms was conducted at the AdSalutem Sleep Institute, which is a reference center specialized in sleep medicine that provides a complete range of the evaluation and treatment of sleep disturbances for children and adults, located in Barcelona, Spain. Between 5 May and 27 November 2023, all consecutive patients who met the eligibility criteria and provided written consent were included in the study. Inclusion criteria were as follows: subjects of both genders, 18 years of age or older, with the presence of one or more insomnia-related symptoms, including difficulty falling asleep, difficulty staying asleep, or waking up too early and not being able to resume sleep. Patients in which insomnia-related symptoms could be caused by other sleep disorders, such as sleep apnea, narcolepsy, circadian rhythm sleep disorders, parasomnia, etc., were excluded from the study. Subjects with a previous diagnosis of DAO enzyme deficiency and following a low-histamine diet or receiving dietary DAO enzyme supplementation were also excluded.

The primary objective of the study was to determine the number (and percentage) of patients with insomnia-related symptoms and DAO enzyme deficiency, defined as the presence of at least one of the four most relevant SNP variants of the *AOC1* gene, including c.691G>7 (rs2052129), c.47C>T (rs10156191), c.995C>T (rs1049742), and c.1990C>G (rs1049793). Secondary objectives included the assessment of DAO deficiency and the types of SNPs in relation to the study variables, including anthropometric data, insomnia-related symptoms, daytime symptoms, and other DAO deficiency-associated symptoms.

The study protocol was approved by the Clinical Research Ethics Committee (CEIC) of Grupo Hospitalario Quirón Cataluña (code DAO-SLEEP-2022, approval date 4 May 2023), Sant Cugat del Vallés, Barcelona, Spain. Written informed consent was obtained from all participants. The study was registered at ClinicalTrials.gov accessed on 5 July 2024 (NCT06488027).

### 2.2. Sample Collection and Genotyping

The four most relevant SNP variants of the *AOC1* gene were measured using the DAO-Test^®^ Genotyping Assay kit (DR Healthcare, Barcelona, Spain). Saliva samples from the oral mucosa were collected by rubbing the inner side of both cheeks using a sterile cotton swab. The genotyping was performed with a Multiplex (Single-Nucleotide Primer Extension) SNuPE (Thermo Fisher Scientific, Applied Biosystems, Waltham, MA, USA) followed by capillary electrophoresis in an ABI 3500 Genetic Analyzer (Thermo Fisher Scientific).

### 2.3. Data Collection

For each participant, the following data were collected: demographic and anthropometric variables; DAO deficiency (defined as the presence of at least one SNP of the *AOC1* gene and categorized as yes/no); the number and distribution of the four variants of the *AOC1* gene; insomnia-related symptoms (trouble falling asleep, staying asleep, early morning awakening, a frequency of insomnia categorized as less than 3 or more than 3 days a week, and daily); daytime repercussions (somnolence, fatigue, cognitive impairment, poor concentration, a lack of attention or memory, mood changes, and headaches); other symptoms (fibromyalgia, migraine, ADHD, allergies or hypersensitivities, gastrointestinal complaints); and sleep medications (Anatomical Therapeutic Chemical [ATC] classification). In addition, a DAO-score based on the number of SNPs was developed, the range of which varied between 0 and 8, where 0 = absence of variants, 1 = heterozygous variant, and 2 = homozygous variant. Therefore, in the case of all four homozygous variants, a DAO-score of 8 is obtained. 

### 2.4. Statistical Analysis

Categorical variables are expressed as frequencies and percentages, and continuous variables as mean and standard deviation (SD) and a 95% confidence interval (CI), or median and interquartile range (IQR) (25th–75th percentile). The chi-square test or Fisher’s exact test were used for the comparison of categorical variables, and Student’s *t* test or the Mann–Whitney *U* test were used for the comparison of quantitative variables according to the conditions of application. Study variables were analyzed in relation to DAO deficiency, DAO-score, and the type and number of SNP variants. A specific analysis of the relationship between c.1990C>G (rs1049793) and the study variables was performed as this is one of the SNPs that has been mostly observed to cause DAO deficiency and the low functionality of histamine metabolism in Caucasian individuals [16,17,18]. Multivariate logistic regression analysis adjusted for age and sex was performed to assess the independent association of genotypic variants and their combinations with specific insomnia-related symptoms, as well as predictive variables of specific genotypic variants. Variables with a *p* value < 0.1 in the univariate analysis were included in the regression models with a stepwise backward selection procedure, and variables with a *p* value > 0.3 were removed. Odds ratios (ORs) and 95% CIs were estimated. Statistical significance was set at *p* < 0.05. The R statistical software package (v4.0.0; R Core Team 2020) was used for the analysis of data.

## 3. Results

### 3.1. General Characteristics of Patients, Prevalence of DAO Deficiency, and SNP Variants

Over the 6-month recruitment period, a total of 167 patients met the inclusion criteria and agreed to participate in the study. There were 50 men and 117 women, with a mean (SD) age of 48.3 (13.5) years, and a mean body mass index (BMI) of 23.8 (4.3) kg/m^2^. Except for the presence of insomnia-related symptoms (which was the reason to seek medical care), subjects were in good general health based on data from the medical history and physical examination.

DAO deficiency was present in 138 patients, with a prevalence rate of 82.6% (95% CI 76–88.1%). In 29 patients (17.4%), no SNPs were found, whereas in the remaining 138 with SNPs, there were 125 (74.9%) heterozygous carriers and 13 (7.8%) homozygous carriers.

The general characteristics of the study population are shown in Table 1. Difficulties in staying asleep were the most common complaint in 88% of patients followed by trouble falling asleep in 60.5%. More than half of patients suffered from insomnia symptoms every day and only 9.6% suffered from them less than 3 days per week. Also, 99.4% reported daytime consequences of insomnia, with fatigue (79.6%), mood changes (72.5%), and poor concentration in 70.1%. Other DAO deficiency-related symptoms included gastrointestinal complaints in 49.1% of cases, respiratory allergies in 25.7%, and migraines in 18.6%. Sleep medications were recorded in 84.9% of subjects, with melatonin alone (24.0%) or in combination with psychotropic drugs (36.5%) being the most common medications. Moreover, 60.5% of patients used over-the-counter sleep aids, 28.1% used benzodiazepines, 27.5% used alpha2-delta ligands, and 10.8% used selective serotonin reuptake inhibitors (SSRIs).

**Figure 1:**
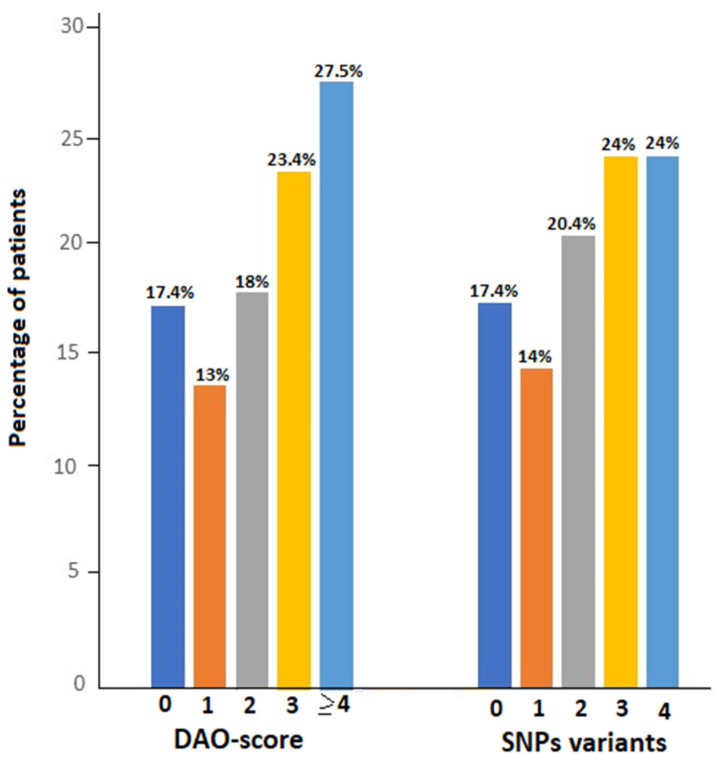
Distribution of DAO-score (0 to ≥4) and number SNP variants (0 to 4) in the study population.

As shown in Figure 1, DAO-score was ≥4 in 46 patients (27.5%), 3 in 39 (23.4%), 2 in 30 (18.0%), and 1 in 23 (13.8%). The number of altered variants was 4 and 3 in 24% of patients, 2 in 34 (20.4%), and 1 in 24 (14.4%). There was a large number of different combinations of SNP variants, with the two most frequent being ‘c.691G>T or c.995C>7 or c.1990C>G’ in 133 patients (79.6%) and ‘c. 691G>T and c.47C>T’ in 93 patients (55.7%) (Table S1, Supplementary Materials).

### 3.2. DAO Deficiency and DAO-Score in Relation to the Study Variables

The distributions of the study variables in the groups of 138 patients with DAO deficiency and 29 patients without DAO deficiency were similar, and there were no statistically significant differences between the groups. However, there were minor non-significant differences in some variables, with patients with a DAO deficiency showing higher percentages of difficulty falling asleep (61.6% vs. 55.2%), early morning awakening (54.3% vs. 51.7%) and reporting a daytime lack of attention or memory (57.2% vs. 51.7%) as compared to those without a DAO deficiency. Food and pharmacological allergies or hypersensitivities showed a trend towards statistical significance, being more frequent among patients with DAO deficiency than in those without DAO deficiency (21.7% vs. 6.9%, *p* = 0.072).

**Figure 2:**
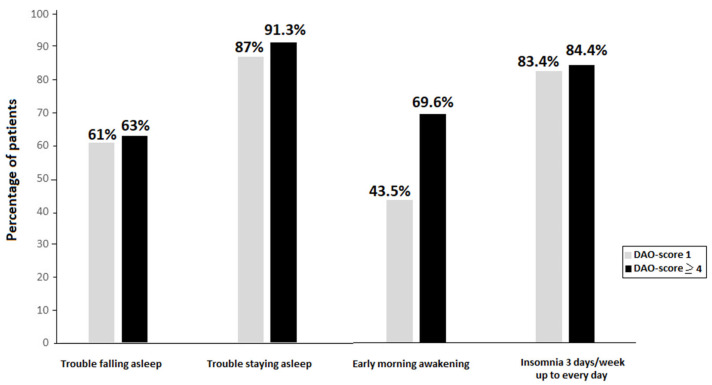
Percentage of patients with nighttime insomnia symptoms according to DAO-score.

In the analysis of insomnia-related symptoms according to the DAO-score, patients with a DAO-score ≥ 4 showed higher percentages of nighttime symptoms than those with a DAO-score of 0, with statistically significant differences in the item of early morning awakenings (69.6 vs. 46.2%, *p* = 0.045). Figure 2 shows the comparison between patients with DAO-score 1 and those with DAO-score ≥ 4 for nighttime insomnia-related symptoms, with somewhat higher percentages among patients with DAO-score ≥ 4.

In relation to daytime repercussions, a lack of attention or memory was more frequent in the group with a DAO-score of 1 than in the group with a DAO-score of 3 (73.9% vs. 46.2%, *p* = 0.038) and mood changes were more frequent in those with a DAO-score of 2 than in those with a DAO-score ≥ 4 (86.7% vs. 60.9%, *p* = 0.019). Also, food and pharmacological allergies–hypersensitivities were more frequent in patients with a DAO-score of 3 than in those with a DAO-score of 0 (28.2% vs. 6.9%, *p* = 0.032). DAO-score was also unrelated to sleep medications, although a higher percentage of patients with a DAO-score ≥ 4 used sleep medications than those with a DAO-score of 0 (91.1% vs. 89.7%, *p* = 1.0) and a DAO-score of 1 (91.1% vs. 82.6%, *p* = 0.428). 

### 3.3. Type and Number of SNP Variants in Relation to the Study Variables

The distribution of genetic variants of the *AOC1* gene according to absence of allelic variants and heterozygous and homozygous SNP carriers did not show statistically significant differences in relation to demographic and anthropometric data, nighttime and daytime repercussions related to insomnia, and other symptoms related to DAO deficiency. However, in the particular case of the c.1990C>G (rs1049793) variant, carriers of c.1990C>G compared to non-carriers showed significantly higher percentages of trouble staying asleep (94.4% vs. 83.3%, *p* = 0.032) and early morning awakening (66.2% vs. 44.8%, *p* = 0.007) (Table 2). Other differences in the remaining study variables were not significant. In the analysis of the combinations of SNP variants, all combinations in which c.1990C>G was present showed higher percentages of patients with early morning awakening than in those in whom this symptom was not present (Table 2).

In relation to the number of SNPs, demographic and anthropometric variables showed a similar distribution among patients with zero, one, two, three, and four variants. Insomnia-related variables also followed a similar pattern, except for early morning awakening which was more frequent in patients with four variants than in those with two variants (70% vs. 44.1%, *p* = 0.038). Other differences in daytime repercussions and other DAO deficiency-related manifestations were not found.

### 3.4. Predictive Variables of Insomnia-Related Symptoms and SNP Variants

In the logistic regression analysis adjusted by age and sex, some SNP variants alone or combined were independent variables significantly associated with some insomnia-related symptoms and manifestations associated with DAO deficiency (Table 3). The combinations of (c.691G>T) and (c.47C>T) and (c.1990C>G) showed an increased risk of trouble staying asleep (OR 4.17) and early morning awakening (OR 3.96). In both cases, each year of increasing age was associated with an increased risk of suffering trouble staying asleep (OR 1.05) and early morning awakening (OR 1.04). In the presence of (c.691G>T) or (c.995C>T), age increased the risk of cognitive impairment (OR 1.05), whereas in the presence of (c.47C>T) and (c.1990C>G), age decreased the risk of mood changes (OR 0.95). Patients with homozygosity in (c.47C>T) had a 30-fold higher risk of ADHD related to DAO deficiency than patients with heterozygosity. Sex had no effect in any of the SNPs.

Likewise, the presence of some clinical manifestations in patients with insomnia were independent predictors of SNPs of the *AOC1* gene. Early morning awakening, dermatological allergies, and food and pharmacological allergies were significantly associated with some specific variants or combinations (Table 4). 

## 4. Discussion

This study, carried out in a consecutive sample of 167 adult patients with insomnia-related symptoms reported in daily practice, shows a high prevalence rate of DAO deficiency of a genetic origin of 82.6%. This finding is clinically relevant as it leaves open the possibility for an alternative treatment strategy of sleep problems based on DAO enzyme supplementation. As far as we are aware, genetic DAO deficiency in patients with insomnia symptoms has not been previously evaluated.

An increase in circulating histamine is a well-known effect of the accumulation of exogenous histamine consumed through diet in the presence of low activity of the DAO enzyme to degrade it. Histamine is involved in numerous physiological functions, and strong and consistent evidence exist to suggest that histamine, acting via H_1_ and/or H_3_ receptors, has a pivotal role in the regulation of sleep–wakefulness [22]. The blockade of the wake promoting effects of histamine via H_1_ receptor antagonism has been a widely used approach to the treatment of insomnia [23,24]. In fact, over half of all over-the-counter sleep aids contain H_1_ receptor antagonists such as diphenhydramine or doxylamine [25]. In the present study, 141 patients (84.9%) took sleep-aiding drugs, and the largest percentage of patients (60.5%) reported using over-the-counter medications.

Early awakening insomnia with an inability to return to sleep is a characteristic symptom of increased histamine levels. In our study, patients with a DAO deficiency showed higher percentages of trouble falling asleep and early awakening as compared with those without a DAO deficiency, although differences were not statistically significant. In relation to daytime repercussions, patients with a DAO deficiency showed higher percentages of fatigue and somnolence. Notably, it was found that the percentage of patients with food and pharmacological allergies was higher among patients with a DAO deficiency (21.7%) as compared to those without a DAO deficiency (6.9%), with the difference showing a trend in statistical significance (*p* = 0.072). Interestingly, when patients were grouped by DAO-score, which reflected the number of heterozygous and homozygous SNP variants, the group with a DAO-score ≥ 4 showed higher percentages of insomnia-related symptoms, in particular, trouble staying asleep and early morning awakening. 

The genetic study was based on the identification of the four SNPs (c.-691G>T, c.47C>T, c.995C>T, and c.1990C>G) more frequently identified in histamine intolerance due to DAO enzyme dysfunction. Data of previous studies of changes in DAO levels of genetic origins in diverse diseases, especially migraines [16], ADHD [17], fibromyalgia [18], and lower urinary tract symptoms (LUTS) [26], are in agreement with the present findings. In all these studies, a high prevalence of genetic DAO deficiency due to these SNPs was found. In a recent retrospective pilot study investigating the prevalence of four variants of the diamine oxidase (DAO) encoding gene (*AOC1*) in a cohort of 100 patients with symptoms of histamine intolerance and 100 healthy individuals, 79% of the individuals with histamine intolerance harbored one or more of the four variants associated with reduced DAO activity [27]. Moreover, it was found that carrying multiple DAO deficiency-associated gene variants and a high load of risk alleles (homozygous) was more relevant than the mere presence of one or more variants [27]. In our study, carriers of the c.1990C>G allele either as a single variant or combined with other alleles as compared to non-carriers showed significantly higher rates of insomnia-related symptoms, especially early morning awakening. In the logistic regression analysis, the presence of c.1990C>G (rs1049793) was also a significant independent risk factor for trouble staying asleep and early awakening, as well as insomnia-related daytime repercussions such as cognitive impairment and a lack or attention or memory, and other symptoms of DAO deficiency such as an allergy or hypersensitivity. 

In patients with migraines and a DAO deficiency, the SNP c.47C>T (rs10156191) was associated with the risk of developing migraines, particularly in women [28]. The SNP c.47C>T was also relevant in our study since, in combination with other variants, it was present as a risk factor for having trouble staying asleep, early awakening, and ADHD. In a prospective cohort of 100 patients with at least moderate LUTS, SNPs of the *AOC1* gene were found in 88% of the patients, with the presence of c.-691G>T and c.47C>T variants associated with a greater severity of obstructive symptoms [26]. In a study of 98 Spanish women with fibromyalgia, the prevalence of genetic DAO deficiency was 74.5% based on the four variants of the *AOC1* gene [18]. SNP deficits were found at frequencies of 53.1% for c.47C>T, 49% for c.691G >T, 48% for c.691G>7, and 19.4% for c.995C>T, which did not differ from the European population [18]. In the present study, somewhat higher percentages of SNPs deficits were found, with 65% for c.691G>7 (rs2052129), 63.5% for c.47C>T (rs10156191), 51.5% for c.995C>T (rs1049742), and 42.5% for c.1990C>G (rs1049793). However, based on the high frequency of the allele frequencies found in patients with insomnia-related symptoms, the genotyping assessment of the *AOC1* gene may constitute a valuable biomarker of DAO enzymatic activity in subjects presenting with sleep problems.

Allergies or hypersensitivities (respiratory, food, pharmacological, and dermatological) and gastrointestinal complaints were also frequently recorded in our study sample. Food and pharmacological allergies/hypersensitivities occurred in 21.7% of patients with a DAO deficiency as compared to 6.9% in those without a DAO deficiency. A plausible association between the DAO activity deficit and allergic rhinitis has been reported in an observational study of 108 patients with symptoms of persistent allergic rhinitis, with a prevalence of 46.3% in those with mild rhinitis and 47.9% in those with moderate/severe symptoms [29]. The authors suggest that allergic rhinitis may be added to other conditions as a potential target of DAO enzyme supplementation [29].

In a recent study in a cohort of 303 children and adolescents with a primary diagnosis of ADHD, 238 patients (78.5%) had at least one of the four SNP variants associated with DAO deficiency [17]. Homozygosity of alleles within rs2052129 and rs10156191 variants related to severe DAO deficiency was associated with a lower intelligence quotient (IQ) and much lower working memory [17]. This interesting observation of *AOC1* gene variants and impairments of cognitive skills in ADHD should be further assessed in other patient populations.

Limitations of the study include the single-center nature of the study, the lack of a control group of healthy subjects without insomnia-related symptoms, and the fact that serum levels of DAO were not measured. Insomnia-related symptoms were assessed on a clinical basis by sleep specialists, but actigraphy studies were not performed, which could be further included in the design of future studies of genetic DAO deficiency in insomnia patients. Our population was in good general health according to data collected from the medical history and physical examination, but work-up studies were not performed. The potential effect of other confounding variables such as the presence of migraines, chronic headaches, or other inflammatory conditions was not evaluated. However, our preliminary real-world study presents novel evidence of a potential link between DAO enzyme deficiency of a genetic origin and clinical symptoms of insomnia. 

## 5. Conclusions

In a consecutive clinical sample of 167 adults presenting with typical nighttime and daytime insomnia-related symptoms, the prevalence of genetic DAO deficiency based on the analysis of the four most relevant SNP variants of the *AOC1* gene was 82.6%. This high prevalence rate of DAO enzyme deficiency in the setting of insomnia warrants confirmation in further studies, but the present results may suggest the potential benefit of DAO supplementation to improve the quality of sleep in these subjects.

## Figures and Tables

**Table 1:** General characteristics of the study subjects.

Variables	Total Subjects, *n* = 167
Gender, *n* (%)	
Men	50 (29.9)
Women	117 (70.1)
Age, years, mean (SD)	48.3 (13.5)
Body mass index (BMI), kg/m^2^, mean (SD)	23.1 (24.5)
DAO deficiency, *n* (%)	138 (82.6)
SNPs genotype, *n* (%)	
No allelic variants	29 (17.4)
Heterozygous carriers	125 (74.9)
Homozygous carriers	13 (7.8)
c.691G>T (rs2052129) GG	58 (34.7)
GT	104 (62.3)
TT	5 (3.0)
c.47C>T (rs10156191) CC	61 (36.5)
CT	98 (58.7)
TT	8 (4.8)
c.959C>T (rs1049742) CC	81 (48.5)
CT	85 (50.9)
TT	1 (0.6)
c.1990C>G (rs1049793) CC	96 (57.5)
CG	67 (40.1)
GG	4 (2.4)
Insomnia-related symptoms, *n* (%)	
Trouble falling asleep	101 (60.5)
Trouble staying asleep	147 (88.0)
Early morning awakening	90 (53.9)
Frequency of symptoms, *n* (%)	
<3 days/week	16 (9.6) *
>3 days/week	63 (38.0)
Every day	87 (52.4)
Daytime symptoms, *n* (%)	166 (99.4)
Somnolence	82 (49.1)
Fatigue	133 (79.6)
Cognitive impairment	17 (10.2)
Poor concentration	117 (70.1)
Lack of attention or memory	94 (56.3)
Mood changes	121 (72.5)
Headache	75 (44.9)
Other symptoms, *n* (%)	129 (77.2)
Fibromyalgia	25 (15.0)
Migraine	31 (18.6)
Attention-deficit hyperactivity disorder (ADHD)	10 (6.0)
Allergies or hypersensitivities, *n* (%)	
Respiratory	43 (25.7)
Alimentary and pharmacological	32 (19.2)
Cutaneous	31 (18.6)

* All these patients were receiving treatment.

**Table 2:** Differences between carriers and non-carriers of the c.1990C>G (rs1049793) variant. and the effect of combinations on early morning awakening.

Variables	c.1990C>G (rs1049793) Variant			Early Morning Awakening		
	Non- Carriers (*n* = 96)	Carriers (*n* = 71)	*p* Value	Absence (*n* = 77)	Presence (*n* = 90)	*p* Value
Trouble staying asleep, *n* (%)						
No	16 (16.7)	4 (5.6)	0.032			
Yes	80 (83.3)	67 (94.4)				
Early morning awakening, *n* (%)						
No	53 (55.2)	24 (33.8)	0.007			
Yes	43 (44.8)	47 (66.2)				
SNPs combinations						
(c.47C>T) & (c.1990C>G)				15 (19.5)	41 (45.6)	0.005
(c.691G>T) & (c.1990C>G)				16 (20.8)	40 (44.4)	0.001
(c.691G>T) & (c.47C>T) & (c.1990C>G)				13 (16.9)	38 (42.2)	0.004
(c.995C>T) & (c.1990C>G)				16 (20.8)	33 (36.7)	0.027
(c.47C>T) & (c.995C>T) & (c.1990C>G)				13 (16.9)	30 (33.3)	0.026
(c.691G>T) & (c.995C>T) & (c.1990C>G)				14 (18.2)	29 (32.2)	0.005
(c.691G>T) & (c.47C>T) & (c.995C>T) & (c.1990C>G)				12 (15.6)	28 (31.1)	0.028

**Table 3:** SNPs as predictors of insomnia-related symptoms and DAO deficiency symptoms.

Predictive Variables of SNPs	Odds Ratio (95% CI)	*p* Value
Trouble staying asleep		
(c.691G>T) & (c.47C>T) & (c.1990C>G)	4.17 (1.25–19.11)	0.0342
(c.691G>T) or (c.47C>T)	0.22 (0.03–0.85)	0.0537
Age (years)	1.05 (1.01–1.09)	0.0184
Sex (male)	1.79 (0.58–6.78)	0.3435
Early morning awakening		
(c.691G>T) & (c.47C>T) & (c.1990C>G)	3.96 (1.90.8.69)	0.0004
Age (years)	1.04 (1.01–1.06)	0.0051
Sex (male)	1.56 (0.76–3.24)	0.2275
Cognitive impairment		
(c.691G>T) or (c.995C>T)	0.39 (0.13–1.13)	0.0753
Age, years	1.05 (1.01–1.09)	0.034
Sex (male)	0.90 (0.26–2.66)	0.8488
Mood changes		
(c.47C>T & (c.1990C>G)	0.66 (0.28–1.48)	0.3329
Age (years)	0.95 (0.92–0.98)	0.0004
Sex (male)	0.61 (0.28–1.32)	0.2042
Attention-deficit hyperactivity disorder (ADHD)		
(c.47C>T) (rs10156191) homozygous vs. heterozygous	29.87 (2.31–760.26)	0.0120
Age (years)	0.98 (0.94–1.03)	0.4953
Sex (male)	3.52 (0.87–15.27)	0.0758

**Table 4:** Predictors of SNPs of the *DAOC1* gene in patients with insomnia.

Predictive Variables	Odds Ratio (95% CI)	*p* Value
Early morning awakening		
(c.1990C>G)	2.20 (1.16–4.25)	0.016
Dermatological allergy		
(c.995G>T)	3.76 (1.19–6.81)	0.021
Food and pharmacological allergies		
(c.691G>T) or (c.47C>T) or (c.995C>T)	4.84 (2.34–31.20)	0.038

## Data Availability

Data supporting the reported results are available from the corresponding author upon request.

## Supplementary Materials

The following supporting information can be downloaded at: https://www.mdpi.com/article/10.3390/jcm13164583/s1, Table S1: Combinations of SNPs variants in the study population.
