# Supplementary material for: Prevalence of Diamine Oxidase Enzyme (DAO) Deficiency in Subjects with Insomnia-Related Symptoms
Source: J Clin Med. 2024 Aug 6;13(16):4583. doi: 10.3390/jcm13164583 (PMC11354289; doi:10.3390/jcm13164583)
Supplement: Supplementary file 1 [file jcm-13-04583-s001.zip › jcm-3127844-supplementary.pdf]

Supplementary Materials

Table S1. Combinations of SNPs variants in the study population

Variables	Total patients, n = 167
Combinations (**OR**), n (%)	
(c.691G>T) or (c.995C>T) or (c.1990C>G)	133 (79.6)
(c.47C>T) or (c.995C>T) or (c.1990C>G)	132 (79.0)
(c.691G>T) or (c.47C>T) or (c.1990C>G)	132 (79.0)
(c.691G>T) or (c.47C>T) or (c.995C>T)	131 (78.4)
(c.691G>T) or (c.1990C>G)	124 (74.3)
(c.691G>T) or (c.995C>T)	124 (74.3)
(c.47C>T) or (c.995C>T)	123 (73.7)
(c.691G>T) or (c.47C>T)	122 (73.1)
(c.47C>T) or (c.1990C>G)	121 (72.5)
(c.995C>T) or (c.1990C>G)	108 (64.7)
Combinations (**AND**), n %	
(c.691G>T) & (c.47C>T)	93 (55.7)
(c.691G>T) & (c.995C>T)	71 (42.5)
(c.47C>T) & (c.995C>T)	69 (41.3)
(c.691G>T) & (c.47C>T) & (c.995C>T)	63 (37.7)
(c.47C>T) & (c.1990C>G)	56 (33.5)
(c.691G>T) & (c.1990C>G)	56 (33.5)
(c.691G>T) & (c.47C>T) & (c.1990C>G)	51 (30.5)
(c.995C>T) & (c.1990C>G)	49 (29.3)
(c.47C>T) & (c.995C>T) & (c.1990C>G)	43 (25.7)
(c.691G>T) & (c.995C>T) & (c.1990C>G)	43 (25.7)
(c.691G>T) & (c.47C>T) & (c.995C>T) & (c.1990C>G)	40 (24.0)
